# Ensuring Quality in Online Palliative Care Resources

**DOI:** 10.3390/cancers8120113

**Published:** 2016-12-13

**Authors:** Jennifer Tieman

**Affiliations:** Discipline of Palliative and Supportive Services, Flinders University, Adelaide, SA 5042, Australia; jennifer.tieman@flinders.edu.au; Tel.: +61-8-7221-8237

**Keywords:** palliative care, end-of-life, knowledge translation, evidence-based practice

## Abstract

Evidence and information is an integral part of the processes enabling clinical and service delivery within health. It is used by health professionals in clinical practice and in developing their professional knowledge, by policy makers in decision making, and is sought by health consumers to help them manage their health needs and assess their options. Increasingly, this evidence and information is being disseminated and sought through online channels. The internet is fundamentally changing how health information is being distributed and accessed. Clinicians, patients, community members, and decision makers have an unprecedented capacity to find online information about palliative care and end-of-life care. However, it is clear that not all individuals have the skills to be able to find and assess the quality of the resources they need. There are also many issues in creating online resources that are current, relevant and authoritative for use by health professionals and by health consumers. This paper explores the processes and structures used in creating a major national palliative care knowledge resource, the CareSearch website, to meet the needs of health professionals and of patients and their families and carers.

## 1. Introduction

Cancer remains a leading cause of death worldwide, accounting for 8.2 million deaths in 2012 with around 14 million new cases being reported in the same year [1]. Lung, liver, stomach, colorectal, breast and oesphogeal cancers were the most common causes of cancer death. New cases are expected to rise by about 70% to 22 million annually over the next two decades.

Palliative care needs to be considered as part of cancer care for many cancers. Indeed the World Health Organisation notes that palliative care can help people live more comfortably and is important for patients in advanced disease where there is little chance of cure Their report suggests that “relief from physical, psychosocial and spiritual problems can be achieved in over 90% of advanced cancer patients through palliative care” [1]. Palliative care can be offered alongside active treatment during advancing disease as well as provided during terminal care delivery in the last days of life and can complement and enhance the care provided to oncology patients [2,3,4,5]. Increasingly the importance of palliative care for non-malignant diseases has also been recognised. Given the growing proportion of older patients and the impact of advancing chronic disease, integrating palliative care alongside chronic care has been noted as an approach to support quality of life and reduce burdensome treatments [6,7,8,9,10].

The evidence base for palliative care as indicated by published research is growing rapidly [11]. More generally, the health and medical knowledge base is also expanding significantly. It is estimated that over 1000 articles are added to PubMed every day, of which 75 are reports of a clinical trial and 11 are systematic reviews [12]. The volume and rate of expansion creates significant issues for health professionals and health systems which struggle to incorporate and utilise the emerging evidence in clinical practice and in service planning.

The problem of an expanding evidence base is compounded by the competence of health professionals’ and policy makers’ ability to search efficiently and effectively for needed literature. Cullen et al.’s study on the information seeking behaviours of junior doctors showed that they had not retained high-level search skills, and lacked skills in identifying and applying best evidence [13]. Similar findings have been reported with respect to nurses’ information seeking skills with authors noting a lack of knowledge about the existence of such sources and limited literature searching skills [14,15,16]. A study of palliative care clinicians searching for palliative care literature on PubMed showed that many struggled to create useful searches. On average only one quarter of the literature known to be relevant was retrieved in the clinicians’ searches [17].

This issue is more critical given that palliative care and oncology both have diverse workforces where evidence from multiple disciplines is likely to be used. The workforce also includes both generalists and specialists across a variety of care settings [18]. For many of these practitioners, palliative care patients will form only part of their patient cohort and hence keeping up to date with the palliative care evidence base is challenging.

The growing evidence base and the need to have access to knowledge both for continuing professional development purposes and for clinical decision making has highlighted the need for knowledge resources that support health professionals. Straus and Haynes have argued for better infrastructure including better knowledge products and tools that are reliable, relevant and readable, and more efficient search strategies [19].

## 2. Methods

### 2.1. Palliative Care Evidence Infrastructure

CareSearch is an online portal funded by the Australian Government Department of Health to provide a palliative care knowledge infrastructure for health professionals and those affected by the need for palliative care, namely, patients, carers, family and friends. The core priority for the CareSearch website (www.caresearch.com.au) is to enable access to palliative care evidence and to evidence-based information. An evidence-based approach is one which turns to the best available evidence to answer clinical and service-related questions. It involves appraising the quality of the evidence being used, and acknowledges the strength of the evidence for decision making while recognising that different groups have different information needs and competencies. The value of research evidence is acknowledged but is contextualised in terms of applicability and suitability for practice. Practice evidence arising from data collection activities is also noted. A number of concepts and principles have driven the design of CareSearch website, including:

- The role of evidence from creation to application, “the knowledge translation cycle”;
- The multidisciplinary nature of palliative care;
- The concept of a palliative care community, that is, both those providing palliative care in any care setting and those affected by the need for palliative care;
- Granularity, or the idea of the size and scale of components and their relationships within a system, that enables users to find specific information and enter at different points within the website;
- Quality processes to ensure trustworthiness and relevance of content, and
- Currency of information being supported by processes that enable information to be regularly updated.

The content covered in the website reflects the information needs of the different users and the multidisciplinary nature of care providers. It also incorporates resources that help professionals find evidence through structured access to indexed and published journal articles and to a web-based collection of unpublished research and literature while enabling them make use of best evidence through clinical evidence pages. PubMed is the key resource utilised as it allows open access searching without registration. Its database characteristics also enable direct hyperlinking of embedded search strategies allowing one click searching of key palliative care topics. A free full text option restricts retrieval to article that the user can read online for free. Research resources including a Research Data Management System assist in the production of research evidence to guide practice while an Education section encourages continuous professional development through information on conferences, short courses and web-based learning. Knowledge Hubs for general practitioners, residential aged care staff, nurses and allied health provide content contextualised for these disciplines and professional groups. Information for patients and families presents content, service information and resources appropriate to the needs of those receiving palliative care to support their choices and decision making.

**Figure 1:**
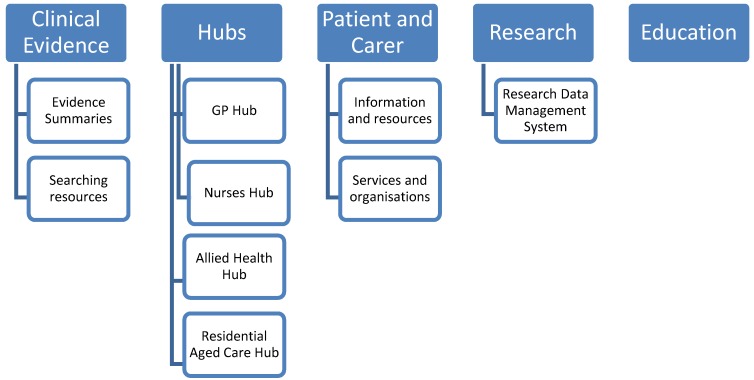
Overview of CareSearch content.

An overview of the structure of the website is outlined in Figure 1.

Although a palliative care website, there are also some cancer specific resources. These include a series of experimentally validated searches for retrieval of lung cancer, non-small cell lung cancer and small cell lung cancer publications. The Review Collection contains specific review sets for brain, breast, colorectal, lung, melanoma, prostate, stomach and general cancer while Life, Hope and Reality provides information for people with advanced cancer, their families, caregivers and friends. More importantly, the website offers significant resources for cancer health professionals to build their understanding and knowledge of palliative care for use within their clinical practice. It also is a trustworthy resource to recommend to patients and families who will need to consider palliative care as part of their care options.

The project has formalised quality processes and utilises a knowledge translation framework [20] that examines the nature and quality of the evidence, considers formats for the audience and the mechanism for delivery, and undertakes monitoring and evaluation processes [21]. Social media strategies are also used to enhance reach and to reinforce evidence messages and ensure ongoing currency.

### 2.2. Governance and Representation

The governance arrangements for CareSearch—as a project and a knowledge translation model—involve multiple layers and a range of different relationships. Central to these arrangements are two national bodies supporting CareSearch—a National Advisory Group that ensures that CareSearch remains closely linked to, and part of, the palliative care sector, and the CareSearch Management Group that provides operational and strategic oversight and make decisions in relation to the CareSearch knowledge model. Membership of the groups represents the diverse constituencies engaging with palliative care issues as well as the professional skill sets required for the delivery of a complex online project. Membership is outlined in Table 1.

Fixed-term and task-focused groups are also formed to meet particular project objectives including the development of significant content or preparation of project communications. There are also a range of other contributors and external roles associated with the project. They include:

- Reviewers where relevant experts volunteer to ensure the quality of content provided through the CareSearch model;
- Project partners ranging from projects dealing with information exchange through cooperation to formal activity collaborations;
- Research collaborations including operational research supports such as the Research Data Management System or offering expertise as part of in-kind support for projects (e.g., sitting on Advisory Groups) as well grant funded research relationships;
- Direct user involvement providing feedback and input on project matters.

The key purpose of the governance arrangements is to ensure the effective operation of CareSearch, to increase awareness of CareSearch and improve outcomes in palliative care, and to enable connections into the sector. We work closely with partner organisations and services to ensure that health professionals are aware of the resource and can provide information to patients and their families. We also seek to have links included in websites and clinical pathways. Search engine optimisation is used to facilitate patient and carer search retrieval of CareSearch.

## 3. Results

### Use and Usefulness

The evaluation framework for the CareSearch website utilises program logic to describe the activities and structures that support the CareSearch knowledge model and the intended consequences for users of CareSearch resources and services, the palliative care sector in general, the health sector more broadly, and for health consumers (patients, carers and families) [22]. The intended consequences or impacts for people who access the CareSearch website and use the information, resources and tools available can be summarised as follows:

- Increased understanding of available CareSearch content and tools;
- Increased understanding about, and positive attitudes towards, evidence and its potential usefulness in practice;
- Increased knowledge about palliative care and related issues;
- Increased knowledge about how to use evidence in practice;
- Intent to change practice.

Monthly website visits show substantial usage with over 100,000 visitors to the site each month. Approximately 60% of page views are of pages intended for health professionals suggesting that clinicians are utilising the evidence resources being made available.

Registrations for newsletters are also an indicator of the use of the website. There are four related project newsletters: @CareSearch (>3300 subscribers), Allied Health News (>1100 subscribers), Residential Aged Care newsletters (>1150 subscribers) and Nurses Hub News (>1950 subscribers). Newsletters not only act as a dissemination vehicle but prompt awareness and use of evidence through the content provided. The Residential Aged Care, Allied Health and Nurses Hub newsletters are all supported by practising clinicians from these disciplines to ensure that content is relevant and useful.

The governance arrangements and stakeholder engagement activities also demonstrate an ongoing commitment from the sector to the project and the project purposes. Stakeholders reported high levels of satisfaction with the governance arrangements [23]. They are not the only performance indicators, however. A series of evaluation studies have been completed that demonstrate positive attitudes to the website, increased understanding of palliative care and the contribution of evidence, and examples of changes in practice [24,25,26,27].

## 4. Discussion

The availability of relevant palliative care evidence that can be accessed when it is needed is a critical part of health’s knowledge infrastructure. Health professionals are classically time poor and the volume of published research can be both hard to find and hard to manage. This may mean that the care being received may not accord with best practice.

For cancer care and palliative care where care needs can be complex and where care provision is multidisciplinary and delivered over a period of time, the health professionals’ information needs are likely to be more extensive. For many patients, palliative care approaches should be an important part of care planning as a result of disease progression. This requires that cancer professionals are familiar with the role of palliative care and comfortable in discussing palliative care considerations with their patients. However, attitudes to, and use of, palliative care services and approaches vary among oncology professionals and cancer services [28,29,30,31]. This is turn may affect the care received by patients and their quality of life in the end stage of the disease.

Resources such as the CareSearch website provide cancer specialists with a single source for palliative care evidence and information to support their own knowledge needs and to facilitate access to palliative care education. It reduces the burdens associated with searching, retrieving ad appraising evidence for its relevance and appropriateness and being online it is available when it is needed. The website also provides options for patients and families making it a “safe” place to recommend to those needing information about palliative care. So for example, a patient recently advised that their cancer has recurred or spread and is no longer curative, can find practical resources and information to support them and their family. This includes information on living with a progressive illness, supports and services for family caregivers, how to deal with financial matters and emotional challenges, and working with health professionals. Information on bereavement, grief and loss is also available to support family and friends.

CareSearch also has a role in building awareness about evidence and about palliative care. By promoting the evidence base for palliative care, it reinforces that palliative care is active care directed to maintaining and improving the quality of life of patients and families.

## 5. Conclusions

Although research driven improvements in prevention and treatment have improved mortality rates within cancer, disease progression means that many patients will still have palliative care needs. Being able to access high quality palliative care resources is an essential part of integrated cancer care. Ongoing currency in terms of the underlying evidence base for practice is fundamental and content should be available when needed and in formats that can be used by the intended audience. Creating such resources is complex requiring input from many different groups and agencies and requires a structured approach to governance, translation and evaluation.

## Figures and Tables

**Table 1:** Governance bodies: representation summary.

National Advisory Group	CareSearch Management Group
Representatives of the following organisations form the National Advisory Group:	Membership skills represented on the CareSearch Management Group:
Advance Care Planning Australia	A representative with policy and systems knowledge
Allied Health Professionals of Australia (AHPA)	A representative with translational science skills
Australian & New Zealand Society Palliative Medicine (ANZSPM)	A representative with skills and knowledge information retrieval and dissemination
Australian Centre for Grief and Bereavement	A consumer representative
Australian College of Rural and Remote Medicine (ACRRM)	Representatives from key academic institutions around Australia
Carers Australia	A representative for bereavement issues
Carer/Consumer Representative	A representative from Palliative Care Australia
Federation of the Ethnic Communities Council of Australia (FECCA)	A representative with IT experience
National Aboriginal Community Controlled Health Organisation (NACCHO)	A representative of Carers Australia
Palliative Care Australia	A representative with legal and business skills
Palliative Care Nurses Australia (PCNA)	A representative of the aged care sector data
Palliative Care Outcomes Collaboration (PCOC)	A representative for Aboriginal and Torres Strait Islander issues
Primary Health Care Research & Information Services (PHCRIS)	A representative of the aged care sector data (Delete data)
Program of Experience in Palliative Care Approach (PEPA/PCC4U)	
Residential Aged Care Sector	
Royal Australian College of General Practitioners (RACGP)	
